# Ambivalent covariance models

**DOI:** 10.1186/s12859-015-0569-1

**Published:** 2015-05-28

**Authors:** Stefan Janssen, Robert Giegerich

**Affiliations:** Practical Computer Science, Faculty of Technology, Bielefeld University, Universitätsstraße 25, Bielefeld, 33615 Germany

**Keywords:** RNA homology search, Covariance model, Consensus structure

## Abstract

**Background:**

Evolutionary variations let us define a set of similar nucleic acid sequences as a family if these different molecules execute a common function. Capturing their sequence variation by using e. g. position specific scoring matrices significantly improves sensitivity of detection tools. Members of a functional (non‐coding) RNA family are affected by these variations not only on the sequence, but also on the structural level. For example, some transfer‐RNAs exhibit a fifth helix in addition to the typical cloverleaf structure. Current covariance models – the unrivaled homology search approach for structured RNA – do not benefit from structural variation within a family, but rather penalize it. This leads to artificial subdivision of families and loss of information in the Rfam database.

**Results:**

We propose an extension to the fundamental architecture of covariance models to allow for several, compatible consensus structures. The resulting models are called *ambivalent* covariance models. Evaluation on several Rfam families shows that coalescence of structural variation within a family by using ambivalent consensus models is superior to subdividing the family into multiple classical covariance models.

**Conclusion:**

A prototype and source code is available at http://bibiserv.cebitec.uni‐bielefeld.de/acms.

**Electronic supplementary material:**

The online version of this article (doi:10.1186/s12859-015-0569-1) contains supplementary material, which is available to authorized users.

## Background

### RNA family modeling in RFAM

The dominating source of information on non‐coding RNA families is the RFAM database [1]. The grouping criterion of RFAM is a follows [2]:

The ideal basis for a new family is an RNA element that has some known functional classification, is evolutionary conserved, and has evidence for a secondary structure.

For many RNA families, this secondary structure is conserved and varies only to a limited degree, such as insertion of unstructured sequence or loss of some base pairs present in the secondary structure. For these families, present‐day modeling techniques work well.

But note that by the above definition, RFAM does not strictly insist on a single common structure, strongly conserved in the above sense. There are well known examples with larger structural variations within a family. The most prominent example is probably the tRNA family (RF00005, RFAM release 10.1). It is known that a minority of the tRNA molecules form a stabilizing “variable loop” in addition to the classical cloverleaf structure. The WIKIPEDIA article on tRNA, which RFAM uses to explain the family, does not fail to point to this fact. The tRNA family comprises 967 members in total, of which a minority of 147 members hold the variable loop. But this “loop” is not just inserted, unstructured sequence, which could be accommodated by present‐day techniques. In contrast to its name, nucleotides of the variable loop form base‐pairs, creating an extra helix which adds stability. A plausible consensus structure for the 147 variable loop members can be constructed by aligning those individual predictions, e. g. with RNAFORESTER [3]. The extra helix is a *bona fide* feature of the tRNA family.

### Limitations of present family modeling

In the presence of structural variation like the one described above, present‐day techniques reach a limit. Family models in RFAM are implemented as covariance models (CMs), constructed by the tool INFERNAL [4]. RFAM constitutes the most important use‐case for covariance models, and in fact, curators of RFAM and developers of INFERNAL do closely cooperate. By construction, INFERNAL’s CMs require all family member sequences to fold into a single, shared secondary consensus structure (*S* *S*_*cons*_) with only small deviations such as indels.

An optional structural feature such as the “variable loop” cannot be accommodated. Replacing in our tRNA example the original *S* *S*_*cons*_ with the variable loop enriched version will have no effect on the CM actually built, because the majority of 820 members has gaps at the variable loop positions and therefore INFERNAL will model those positions as insertions. In effect, the introduced variable loop sub‐structure is taken out off *S* *S*_*cons*_, and informative covariance from base‐pairs of this extra helix (where present) cannot be captured.

One might also consider enforcing a five‐helix *S* *S*_*cons*_ by increasing the allowed gap ratio. Base pairs in the minority structures could now contribute. But this idea would impose large deletion costs on the 820 majority members when aligning to the new model. In either case, an improvement can not be reached by providing an alternative alignment and a single *S* *S*_*cons*_.

### Allowing for structural variation in RNA families

The architecture of CMs has been remarkably stable for 20 years, serving its purpose very effectively. But for accommodating multiple structures, as shown above, one must extend the classical definition and construction of CMs. With the introduction of *ambivalent* Covariance Models (aCMs), we provide such a generalization.

An aCM is a CM constructed from several consensus structures, allowing sequences to fold into a set of predefined alternatives without penalty, and exploiting sequence conservation and covariance at all points.

The organization of this contribution is as follows: We briefly recapitulate the technical background of CMs and their INFERNAL implementation in Section ‘Introduction:classical covariance models’. The classical architecture is hard‐wired in INFERNAL’s CMs. To be able to modify it, we first re‐create classical CMs in a rapid prototyping framework in Section ‘Recreating the core of INFERNAL’, where the model generation process can be described on the abstraction level of context‐free grammars. Extending these grammars by extra rules that branch between alternative consensus structures, we arrive at aCMs in Section ‘Ambivalent covariance models’. Section ‘Evaluation’ is devoted to their evaluation, comparing ambivalent models to classical models in the presence of structural variation.

### Recent alternative approaches to structural variability in RNA

While our work is firmly based on the classical work on CMs, there are other interesting approaches, not based on CMs, which address structural variation in related RNA sequences. Both approaches are very recent and have appeared while this manuscript was in preparation.

Saffarian *et al.* suggest a combinatorial approach to search for a predefined set of alternative structures, called *multi‐structures*, in RNA sequences [5]. They describe this set as a formal grammar, on the granularity of a predefined set of stable helices. This can be used to describe RNA families with structural variation, but also interesting suboptimal structures from the search space of the same type of RNA. We will refer to an aspect of this work in Section ‘Ambivalent covariance models’.

Reinkensmeier and Giegerich elaborate the approach of *thermodynamic machters* to define the “cuckoo” RNA family [6]. The characteristic cuckoo motif consists of 2 ‐ 4 hairpins with no sequence conservation in the helices, but exhibiting a conserved loop motif. Their approach is based on the theromodynamic energy model, but in principle, the energy rules can be replaced by stochastic scoring, taking the approach closer to (extended) CMs. Although also based on formal grammars, the approach is semi‐automatic, allowing the model designer careful tuning of the generated matchers, which can be considered a blessing as well as a burden.

## Introduction: classical covariance models

A *covariance model* (CM) is a stochastic approach to quantify homology of an RNA sequence to a *family* of sequences. The family consists of an aligned set of RNA sequences (*MSA*), which are believed to share the same functionality, shape or other grouping properties, together with one (pseudoknot free) consensus secondary structure (*S* *S*_*cons*_). The machinery follows the Bayesian interpretation of probabilities, by updating family independent expert knowledge (priors) with those frequencies observed in the given family (posteriors). Amalgamation of priors and posteriors is called “training” and has to be done just once. Result of the training is a *stochastic context free grammar* (SCFG), whose production rules are augmented with transition and emission‐probabilities. The *architecture* of CMs can be described by an *architecture grammar*, which is a grammar that can parse any RNA secondary structure. The family model grammar is generated from the architecture grammar by specializing it to *S* *S*_*cons*_ (for a tutorial exposition of this view see [7]), and its parameters are trained from the multiple alignment.

Covariance models follow the principles of *Hidden Markov Models* (HMMs) [8], but are more powerful in order to account for distant but coupled positions, which represent the base‐pairs of *S* *S*_*cons*_. While an HMM runs through a linear sequence of states, the transition graph of the CM “automaton” has a tree‐like branching structure that mimics *S* *S*_*cons*_. For historical reasons, the classical description of this technique [4] uses a mixture of HMM (“state transition”) and grammar terminology (“bifurcation rule”); here we try to stick to the latter, providing translation of terminology where appropriate.

The parses constructed by the family model grammar for any sequence are very similar. Abstracting away insertions and deletions, they all indicate the same structure, namely the given *S* *S*_*cons*_. This unique abstract parse tree is called a “guide tree” in INFERNAL terminology. When we apply the CM to an RNA sequence, we can obtain the probability of the most likely parse of the given sequence. Since this probability tends to be very small, it is scaled to a background model. This is done in terms of a log odds ratio, which provides a bit‐score, which finally expresses homology between sequence and family. It is up to the user to decide if the bit‐score suffices to accept the input sequence as a new family member.

### The INFERNAL software suite

#### Reference implementation

The INFERNAL software package [9], a product of 20 years of careful software engineering, is *the* reference implementation of CMs. It does not only provide programs for the above described tasks of training (CMBUILD) and searching (CMSEARCH), but e. g. also tools to “calibrate” the CM – basically to provide E‐ and P‐values for the bit‐scores – or to align the new member to the existing family.

Scoring a sequence *s* of length *n* to a family model of length *m* takes *O*(*n*∗*m* ^3^) time and *O*(*n*∗*m* ^2^) space. Such high computational cost arises from parsing with a context free grammar which itself has a size proportional to the length of the input. Much of the efforts spent in the past twenty years aimed to lower these high computational demands. In 2002, Eddy introduced a memory‐efficient divide‐and‐conquer variant of the CYK (Cocke‐Younger‐Kasami) algorithm [8,10]. An HMM pre‐filtering strategy was pioneered by Weinberg and Ruzzo in 2006 [11]. Since 2007, remaining candidate sequences of *s* are scored with a heuristics, called “query dependent banding” [12], which tightly restricts the search space while hopefully retaining the most likely candidate. Wherever possible, INFERNAL uses parallelization to further speed up the run‐time. For all these improvements, the basic architecture of CMs remained untouched. Furthermore, statistics are enhanced for training by sequence weighting and expectation maximization. Sequence weighting is to adjust for potential sub‐grouping of the family members. Expectation maximization should compensate for over‐fitting, by finding a suitable trade‐off between priors and posteriors. CMSEARCH can be run in glocal (aka “small in large” or “free shift”) or local mode. The reported bit‐score might be either the probability of the most likely state‐path (the CYK algorithm, which is the analogue to the “Viterbi” algorithm in HMMs), or probability sum of all possible state paths (“inside” algorithm; HMM analogue is called “forward”) [8].

#### Model construction

We review the model construction process in some detail, as we are going to re‐implement it from scratch before extending it.

**Figure 1:**
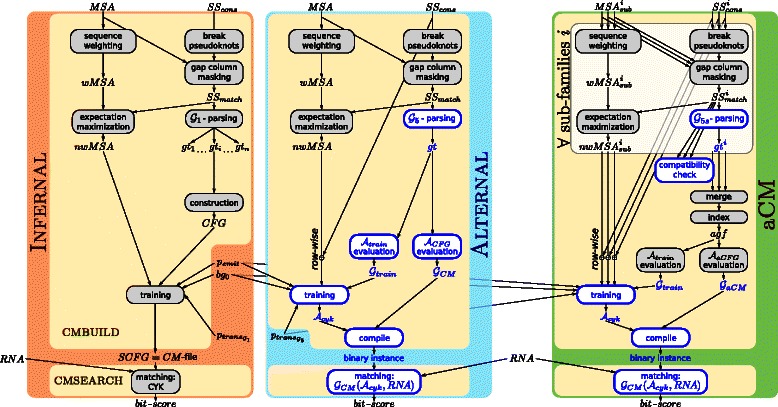
Flowgrams of INFERNAL (red, discussed here), our ADP re‐implementation ALTERNAL (blue, explained in Section ‘Recreating the core of INFERNAL’) and the extension to ambivalent consensus structures aCM (green, introduced in Section ‘Ambivalent covariance models’). Input is the multiple sequence alignment(s) *MSA* and consensus structure(s) *S* *S*_*cons*_ for the construction of a model and *RNA* for homology search. Blue colored items are ADP components like grammars or algebras. The white box in aCM shall indicate that those operations are performed for each sub‐family.

Before a query RNA sequence can be assessed for homology to a family of interest, a covariance model (CM) must first be built from the given family information, namely the multiple sequence alignment (*MSA* of *k* sequences) and the single consensus secondary structure (*S* *S*_*cons*_). This construction task is carried out by the program CMBUILD of the INFERNAL package (see the reddish box in Figure 1). CMBUILD takes *MSA* and *S* *S*_*cons*_ as its inputs and finally produces a CM encoded as a table in a flat‐file. This table encodes the parser for the family model grammar, while the underlying family model grammar is not constructed explicitly. (However, it can be extracted from the file by upward compilation [13]).

The complete procedure can be divided into the following seven sub‐processes:

1. **Break pseudoknots**: CMs cannot cope with pseudoknots due to the restriction to context free grammars. However, some Rfam families (89 of 2,208 in release 11.0) contain annotations for crossing base‐pairs. One of the crossing pairs is annotated by standard brackets in *S* *S*_*cons*_, the other with upper‐ and lower‐ case letters for the opening and closing partner, respectively. All bracket annotated pairs must either be nested or adjacent to all other bracket annotated pairs. To obtain a pseudoknot free structure, all letters are simply converted into unpaired bases.
2. **Gap column masking**: Integrating a further sequence into a *MSA* with an insertion inevitably causes new columns which hold gaps for the majority of sequences. To counteract this effect, Infernal tells apart “matching”‐columns with a gap‐ratio below a certain threshold (50% by default) and “insertion”‐columns above this threshold. Only the first column‐type is reflected in the final architecture of the CM. The structure *S* *S*_*match*_ is *S* *S*_*cons*_, where “insertion”‐positions are removed. If both columns of a base‐pair fall into different column‐types, the pair must be broken and only the partner below the threshold appears in *S* *S*_*match*_ as an unpaired base.
3. $\boldsymbol{\mathcal {G}}_{1}$ **‐parsing**: To generate the family model SCFG, *S* *S*_*match*_ is parsed with the architecture grammar $\mathcal {G}_{1}$ to gain a guide‐tree (*gt*) as an intermediate step. In grammar terminology, this guide tree is the only parse tree which the family model grammar can generate without using rules for insertion or deletion. The architecture grammar $\mathcal {G}_{1}$ is shown in Figure 2. (Since the architecture grammar $\mathcal {G}_{1}$ to parse the structure is syntactically ambiguous [14], the result is not unique. One of the parses must be chosen as the guide tree according to a specific set of objectives. The online Additional file 1 informs about these objectives in detail.)

**Figure 2:**
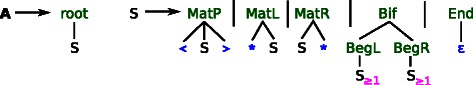
Architecture grammar $\mathcal {G}_{1}$ to parse *S* *S*_*match*_ to generate INFERNAL style CMs. The axiom is A. Terminal symbols are colored blue, algebra functions green and filters magenta. Terminal *ε* is the empty word; < and > denotes the opening and closing bases of a pair and * is the unpaired base. The magenta subscript ≥1 requires the affected non‐terminals to parse at least one character from the input, i. e. the branches of a bifurcation cannot be empty.
4. **Construction**: The family model grammar is now extended with productions providing for insertions in the query sequence. These are always considered unpaired bases. And it is extended by productions providing for deletions from *S* *S*_*cons*_, where unpaired bases as well as paired bases can disappear.

The result of at this point is a the family model grammar, which reflects the unknotted, “insertion”‐column masked consensus structure *S* *S*_*match*_. To turn this grammar into a CM, all transitions and emissions must be associated with probabilities, which are inferred from the *MSA* via the following processes:

5. **Sequence weighting**: The *MSA* might be subdivided into several sub‐groups. Due to different biological interests, different levels of experimental difficulties or other reasons, the *MSA* might have many representatives for one sub‐group, but only a few for the other. Since the CM should be able to detect both sub‐groups with equal strength, Infernal uses different weights for training with the *MSA* sequences of both sub‐groups. Version 1.0.2 of Infernal uses the “Gerstein / Sonnhammer / Chothia tree weights” [15] to turn a *MSA* into a weighted alignment (*wMSA*).
6. **Expectation maximization**: In order to avoid overfitting, Infernal uses a rudimentary expectation maximization process to balance the influence of priors and posteriors. The theoretical background is given in [16] and its application in Infernal in [17]. Taking only the “matching”‐columns of *wMSA* into account, given by *S* *S*_*match*_, sequence weights are re‐**n**ormalized to a value *k*_*eff*_≤*k* to form *nwMSA*. The value *k*_*eff*_ is the result of an optimization problem, where the alignment entropy shall approximate a pre‐defined “target” value.
7. **Training**: Finally, the *MSA* is used to count how often which production rules of the family model grammar are used in parsing the data and which nucleotide are produced by the terminal symbols in the grammar. The occurrences do not contribute equally, but are weighted according to *nwMSA*. Priors for emissions *p*_*emit*_ and transitions in a $\mathcal {G}_{1}$ model $p_{{trans}_{\mathcal {G}_{1}}}$ are added to the counts before probabilities are computed. Furthermore, emission probabilities are contrasted with a simple background model *b* *g*_0_, where unpaired and paired emissions are equally distributed. There is no background model for the transitions. The resulting *SCFG* is saved in a CM‐file, which encodes the parser for the stochastic family model grammar in a tabular form.

The program CMSEARCH of INFERNAL reads the model from the CM‐file, takes a query RNA sequence as second input and computes the CYK bit‐score for matching the sequence against the model: Process **matching CYK** in Figure 1.

Our generalization of CMs to aCMs touches the core aspects of this construction process. In particular, we will have to change model architecture and family model grammar. Presently, they are concepts which explain the approach, but are not constituents of the INFERNAL software or its output that we can get our hands on, take out and change. Hence, we re‐create INFERNAL in a more flexible framework, in order to venture on to our extension.

## Recreating the core of INFERNAL

### Overview

As the first step to our extension towards aCMs, we will produce a software named ALTERNAL, which re‐implements the INFERNAL approach, but with a different architecture and an explicit construction of the family model grammars. As the construction of the family model grammar entails parsing *S* *S*_*cons*_ with the architecture grammar, and running the family model means dynamic programming, a programming system that combines both techniques on a high level of abstraction is highly useful for our effort.

In this section, we first review the technique of algebraic dynamic programming, and use it to reconstruct INFERNAL in a way that we can extend towards aCMs in Section ‘Ambivalent covariance models’. Asymptotic run‐times in ALTERNAL remain the same as reported for Infernal, e.g. scoring a sequence *s* of length *n* to a family model of length *m* takes *O*(*n*∗*m* ^3^) time and *O*(*n*∗*m* ^2^) space. The speed‐up techniques provided with INFERNAL are not re‐implemented. Please keep in mind that the software ALTERNAL serves as an intermediate step; it is not intended to go out and compete with INFERNAL.

### ADP and the BELLMAN’S GAP system

Algebraic Dynamic Programming (ADP) [18] is a discipline to formulate algorithms for sequential problems. Its high level of abstraction allows for a clear separation of concerns. (i) A combinatorial search space is generated by a *regular tree grammar* . (ii) Each candidate of the search space is evaluated by an *evaluation algebra* . (iii) The “best” candidate is determined by an *objective function*.

Evaluating a search space described by grammar  and input sequence *x*, using evaluation algebra , is simply denoted by

$$\mathcal{G}(\mathcal{A}, x). $$

Normally, this will return the optimal candidate in the search space. When  is the family model grammar,  the stochastic scoring algebra, and *x* the query sequence, we obtain the best alignment of the query to the model. But the search space may be small and the “score” need not be a number: When  is the *architecture grammar*, *x* is some *S* *S*_*match*_, and the scoring functions in  compute grammar rules, the resulting “score” will be the family model grammar. Wait and see.

In ADP, components of a problem specification can be easily replaced or even combined in algebra products [19] to tackle new challenges without low‐level reprogramming. For a detailed exposition of the formal concepts of ADP see [18]. In our experience, when one has to modify a nontrivial dynamic programming algorithm, it is often easier to re‐implement it in ADP rather than to tinker with the existing source code.

The BELLMAN’S GAP system is a recent implementation of ADP, which we rely on for the present project [20]. Covariance model generation as well as application are formulated in the language GAP‐L, and translated into C++ code by the GAP‐C compiler.

The software ALTERNAL (blue box in Figure 1) is our re‐implementation of INFERNAL with BELLMAN’S GAP, where many processes are replaced by ADP versions (blue font/borders). It mimics INFERNAL as described in Section ‘Model construction’, but comes with two fundamental modifications: First, the replacement of the architecture grammar and second the way to obtain the counts for training. Notably, the search spaces of CMs, either created by INFERNAL or by ALTERNAL, are identical. Both implementations will consider the same possible alignments of a query sequence to the model. In principle, this should be true for the bit‐scores of the search process as well. The processes *break pseudoknots*, *gap column masking*, *sequence weighting* and *expectation maximization* remain untouched for the moment.

### Change 1: replacing the architecture grammar

**Figure 3:**
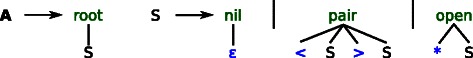
Architecture grammar $\mathcal {G}_{5}$ to parse *S* *S*_*match*_ to generate ALTERNAL style CMs. Here, the *ϵ* terminal parser in ADP returns the input position where the empty word was recognized, for eventual use in other evaluation functions.

**Figure 4:**
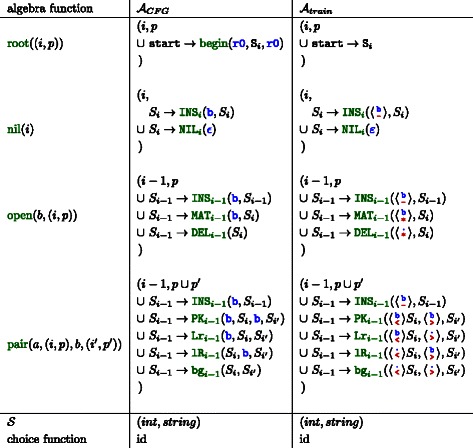
Evaluation algebras $\mathcal {A}_{\textit {CFG}}$ and $\mathcal {A}_{\textit {train}}$ to generate family specific grammars for homology search and model training. Non‐terminals and algebra functions of the generated grammars must be indexed by their position in *S* *S*_*match*_. The position is provided by the special terminal parser of nil and propagated to all other algebra functions via the first component of the return type. The second component holds a string representation of the generated grammar rules.

The architecture grammar of INFERNAL is $\mathcal {G}_{1}$, depicted in Figure 2. For ALTERNAL, we replace the architecture grammar by $\mathcal {G}_{5}$, see Figure 3. The advantage is three fold: First, it is guaranteed that the $\mathcal {G}_{5}$‐parsing process of *S* *S*_*match*_ will exactly yield one parse (guide tree in INFERNAL terminology). Selecting a specific one from several alternatives by *ad‐hoc* criteria is avoided. Second, the *construction*‐process in ALTERNAL can be described by evaluating *S* *S*_*cons*_ with the evaluation algebra $\mathcal {A}_{\textit {CFG}}$ (see Figure 4). The “scoring” functions of this algebra $\mathcal {A}_{\textit {CFG}}$ do not compute scores. Rather, they compute the productions that make up the family model grammar. And third, compared to a $\mathcal {G}_{1}$ architecture CM, the number of production rules is roughly reduced four‐fold in a $\mathcal {G}_{5}$ architecture (see Additional file 1 for details), while exactly keeping the same search space. Thus, the training has to infer fewer parameters. The result of the *A*_*CFG*_‐*evaluation* is another grammar — the family model grammar $\mathcal {G}_{\textit {CM}}$ which captures the family specific architecture of the CFG:

$$\mathcal{G}_{CM} = \mathcal{G}_{5}(\mathcal{A}_{CFG}, {SS}_{match}). $$

**Figure 5:**
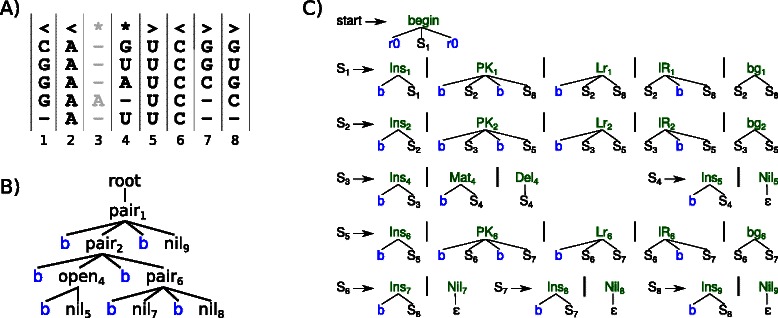
Example for ALTERNAL CMs: Input *MSA* and *S* *S*_*cons*_ is given in **A**. The guide‐tree for *S* *S*_*match*_ is shown in **B**. The $\mathcal {G}_{5}$‐style CFG of the model is shown in **C**. Terminal parser b reads one base from the input sequence, while r0 consumes a potentially empty region of consecutive bases. The first rule facilitates a glocal alignment.

An example is given in Figure 5. Panel A) shows the *MSA* with *k*=5 sequences. The consensus structure *S* *S*_*cons*_ is given as the top row. The third column is shaded, because it holds more gaps than bases and will be modeled as an insertion. Due to this gap, *S* *S*_*match*_ is <<∗><>>, which is different from the initial *S* *S*_*cons*_. The single guide‐tree which results from parsing *S* *S*_*match*_ with the architecture grammar $\mathcal {G}_{5}$ is given in Panel B) of Figure 5. Panel C) presents the final CM grammar, which is the result of evaluating the guide‐tree with $\mathcal {A}_{\textit {CFG}}$. See the online Additional file 1 for further details.

### Change 2: gaining posteriors

Once the architecture of a CM is fixed, posteriors from the *MSA* can be inferred. Aligning a query sequence to a model becomes an optimization problem over many different derivations, because we do not know the best situation relative to the consensus structure for each nucleotide in advance (This is the best scoring parse, called *state path* in HMM terminology). But we do know the single correct derivation for training, because the training sequences are already aligned. And thus, we have only one candidate in the search space if we force a (training) sequence into a concrete derivation. We can do so by providing this derivation as a second input, besides the nucleotide sequence itself. We need a third, family specific ADP grammar $\mathcal {G}_{\textit {train}}$ to simultaneously parse one *MSA* row and a secondary structure. This grammar is created by evaluating *gt* again, but this time with the evaluation algebra $\mathcal {A}_{\textit {train}}$ (see Figure 4):

$$\mathcal{G}_{train} = \mathcal{G}_{5}(A_{train}, {SS}_{match}). $$

To gain the desired counts in the training process, every *MSA* row must be parsed together with ${SS}_{{train}_{k}}\phantom {\dot {i}\!}$ which is a slightly modified version of *S* *S*_*cons*_: If according columns are marked as “insertion‐columns”, unpaired positions become gaps, and partnering positions become unpaired bases. Mapping a single row of *MSA* to ${SS}_{{train}_{k}}\phantom {\dot {i}\!}$ might produce columns consisting of a gap in sequence *and* model: $\langle {\dot {\text {{\tt {-}}}}}\rangle $. These columns must be removed from both inputs. This mapping shall be indicated by the ⊕‐operator in Figure 1.

An enumeration algebra $\mathcal {A}_{\textit {enum}}$ is a generic representation of candidates of the search space, which can automatically be produced by the BELLMAN’S GAP compiler. It records which algebra functions were called with which parts of the inputs, but gives no hint about the used non‐terminals, i. e. the grammar production rules. The trace of the algebra functions is exactly what we need for training, because they give rise to the derivation through the CM and the sub‐words of the nucleotide input, i. e. single bases inform us about the concrete emissions. By creating enum‐representations for all training pairs, consisting of the *MSA* rows and ${SS}_{{train}_{k}}\phantom {\dot {i}\!}$, and accumulating the occurrence count of each algebra function, we simply get the desired counts. As in INFERNAL, counts do not equally contribute, but are weighted according to *nwMSA*. The resulting probabilities of this training process will be used to generate a family specific CYK algebra $\mathcal {A}_{\textit {CYK}}$.

Due to our shift to a $\mathcal {G}_{5}$ architecture in ALTERNAL, we cannot re‐use the priors of INFERNAL for the $\mathcal {G}_{1}$ transitions $p_{{trans}_{\mathcal {G}_{1}}}$. Instead, we once need to derive a set of transition priors $p_{{trans}_{\mathcal {G}_{5}}}$ from a trusted set of families. We do so with the same set as used for INFERNAL, which is described in [12].

Finally, for the search process, a query RNA sequence *x* can now be parsed by $\mathcal {G}_{\textit {CM}}$. With $\mathcal {G}_{\textit {CM}}$ and $\mathcal {A}_{\textit {CYK}}$ coded in GAP‐L, BELLMAN’S GAP will compile this into a program instance for the call of $\mathcal {G}_{\textit {CM}}(\mathcal {A}_{\textit {CYK}}, x)$ for any *x*. All candidates of the search space will be evaluated by $\mathcal {A}_{\textit {CYK}}$ and the maximal score will be reported.

### Approving faithful re‐implementation

To approve that ALTERNAL is a faithful remake of INFERNAL, we must show that it considers the same search space and scores candidates in a similar way.

To verify our statement that the search spaces of INFERNAL and ALTERNAL are identical, we performed the following experiment: The CM flat files, as the product of CMBUILD from the INFERNAL package, contain the models $\mathcal {G}_{1}$‐architecture as well as all transition and emission probabilities. The upward compiler described in [13] can parse these files and construct an equivalent ADP grammar. We use the same idea to produce grammars $\mathcal {G}_{\textit {CM}}^{\mathcal {G}_{1}}$ coded in GAP‐L. Again, a counting algebra $\mathcal {A}_{\textit {count}}$ can automatically be generated by BELLMAN’S GAP. Thus, we can easily determine and compare the search spaces sizes of $\mathcal {G}_{\textit {CM}}^{\mathcal {G}_{1}}(\mathcal {A}_{\textit {count}}, r)$ and $\mathcal {G}_{\textit {CM}}(\mathcal {A}_{\textit {count}}, r)$. In fact, they are in all test cases identical (data not shown). To proof that the upward compiled versions are identical to INFERNAL, we measured a shift of bitscores and found that the median shift is 0.000 for “original” and “shuffled” sequences.

**Figure 6:**
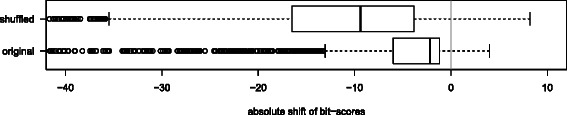
Bit‐shift of global CYK scores between INFERNAL and ALTERNAL for positive (original) and negative (shuffled) test cases in terms of absolute difference of CYK bit‐scores. For example, a sequence scoring 40 bits with INFERNAL and 44 bits with ALTERNAL would have a bit‐shift of +4.

To access the capability of ALTERNAL to reproduce the scoring results of INFERNAL, we set‐up the following experiment for each of the 2,208 families in the RFAM 11.0 release: First, a traditional CM is generated from the RFAM seed alignment *MSA* which includes the consensus structure *S* *S*_*cons*_ with the CMBUILD software (version 1.0.2) using default parameters. Next, a single “original” row from *MSA* is randomly chosen, gaps are removed and the thus gap‐freed sequence is globally aligned to the previously built CM with the program CMALIGN (version 1.0.2) from the INFERNAL package with parameters set to –no-null3 -1 –cyk –nonbanded. The resulting bit‐score constitutes our base‐line. We prefer CMALIGN over CMSEARCH at this step, because only the first one is able to compute global alignments. Another base‐line score for negative test cases is computed by aligning a di‐nucleotide “shuffled” (by the program USHUFFLE [21]) version of the previously used sequence to the same CM. Second, we compile a $\mathcal {G}_{\textit {CM}}(\mathcal {A}_{\textit {CYK}}, r)$ instance with ALTERNAL for the family and run this instance with the exact same two sequences *r* (original and shuffled) as used before. By subtracting the base‐line value from the bit‐score computed by $\mathcal {G}_{\textit {CM}}(\mathcal {A}_{\textit {CYK}}, r)$ we obtain the bit‐shift between INFERNAL and ALTERNAL. The bit‐shifts for all 2,208 families are depicted as boxplots in Figure 6. Except for those seven families where *S* *S*_*match*_ exceeds 750 bases which causes memory overflows.

We observe small but significant shifts of bit‐scores between the two programs, which cannot be explained by rounding errors. Positive test cases show a median shift of −2.2 bits, negative ones a shift of −9.4 bits. If we force both programs to separately report bit‐scores originating from emissions and transitions, we see that these differences only stem from shifts in the transitions and not from emissions (data not shown). The reason is the change of the underlying architecture from $\mathcal {G}_{1}$ to $\mathcal {G}_{5}$. In consequence, building two CMs from the same family leads to two fundamentally different grammars, which still have the same semantic meaning. Emissions are not affected by this change, they are only associated at different non‐terminals. But there is no possible mapping for the transitions between those two models, which causes divergent posteriors at training and different numbers and values of single transitions for parses of those models at the alignment step.

**Figure 7:**
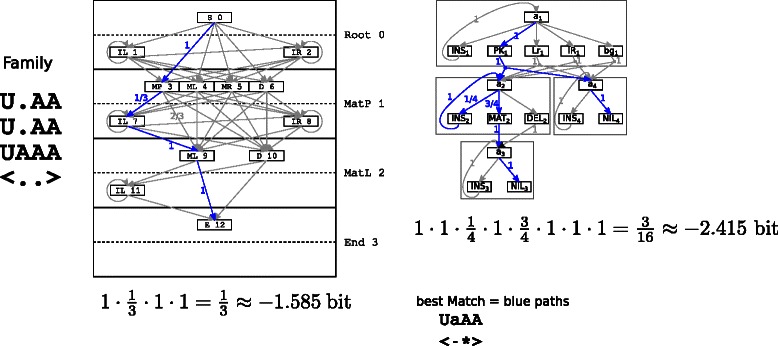
Impact of different model architectures on transition caused bit‐scores. The family is translated into CMs of the $\mathcal {G}_{1}$ type (left) and $\mathcal {G}_{5}$ (right). Aligning the same sequence results in different scores even if the alignment have the same semantic meaning, e. g. the outer bases U and A match the pair, a is an insertion relative to the model and the second to last A matches the unpaired position.

Figure 7 shows an illustrative example. The same tiny family with three sequences in a four‐column alignment and a simple hairpin as consensus structure is translated into a $\mathcal {G}_{1}$ model (on the left) and a $\mathcal {G}_{5}$ model (on the right). Let us assume that the highest scoring alignment for both models would be $\left \langle {\begin {array}{ll}{\text {\tt {UaAA}}}\\\text {\tt {{<}{-}}}\!*\!\text {\tt {>}}\end {array}}\right \rangle $. The corresponding parses are coloured blue in both models. While $\mathcal {G}_{1}$ requires only four transitions, the parse of $\mathcal {G}_{5}$ needs twice as many, also with different values. Thus, the alignment with the exact same meaning gains −1.585 bits from $\mathcal {G}_{1}$ and only −2.415 bits from $\mathcal {G}_{5}$.

The tendency of $\mathcal {G}_{5}$ for more single transitions results in lower bit‐scores as shown in Figure 6. Fortunately, this trend hits negative cases harder than positive ones, which means that $\mathcal {G}_{5}$ models have a stronger discriminative power.

Thus, we conclude that ALTERNAL is a faithful re‐implementation of INFERNAL, close enough such that the improvements achieved by our pending generalization can also be expected from analog extensions to INFERNAL.

## Ambivalent covariance models

We must define aCMs, specify the input for aCM construction, and describe the generation process. The evaluation of the new approach will be given in Section ‘Evaluation’.

### Problem definition and input format

aCMs extend classical CMs by modeling for RNA sequence families that fold into a predefined set of consensus structures. Mathematically, aCMs are stochastic context free grammars, generated from a $\mathcal {G}_{5}$ architecture grammar, and with parameters trained as with classical CMs.

The input to aCM construction is a multiple RNA sequence alignment in Stockholm format, which indicates several consensus structures. The creator arranges the alignment rows according to different structural features and supplies one consensus structure for each sub‐family. To distinguish the *n* different sub‐families in the input file, one must prefix the sequence names and the multiple SS_cons lines for the consensus structures with an arbitrary sub‐family name, followed by an “@” delimiter character. Here, for the purpose of exposition, we use colours instead.

**Figure 8:**
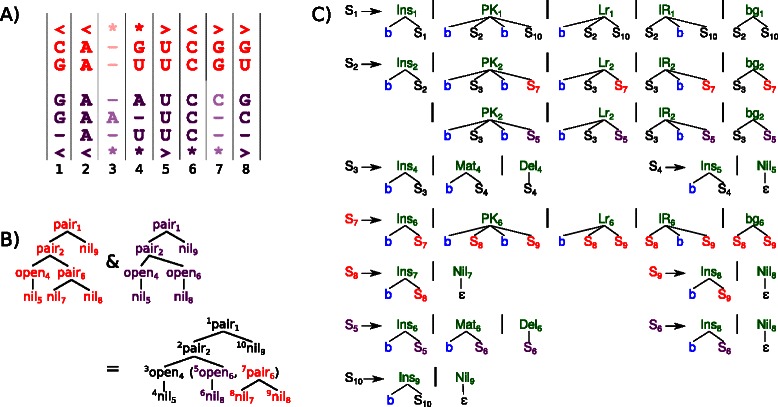
Example for aCMs: Input *MSA* is split into red and purple sub‐families. Their consensus structures are given as first and last row in **A**. The individual guide trees for red and purple and their ambivalent guide forest are shown in **B**. For simplicity, we omit terminal symbols for bases and skip‐nodes – described below – in the trees. That is why subscript 3, pointing to the “insertion”‐column, does not appear in the guide trees. The $\mathcal {G}_{5}$‐style CFG of the ambivalent model is shown in **C**.

Let us re‐use the previous example alignment of Figure 5, which is here sub‐divided into the red sub‐family of the first two sequences and a purple sub‐family holding the remaining three sequences (*n*=2), see Panel A) in Figure 8. The consensus structure for the red sub‐family ${SS}_{\textit {cons}}^{red}$ remains unchanged. To honor the mutations in column 7 for the purple sub‐family, the consensus structure ${SS}_{\textit {cons}}^{purple}$ is slightly different, i. e. the base‐pair is converted into two unpaired bases.

The *S* *S*_*cons*_ rows in the alignment must represent a correct multiple *structure* alignment. This condition is not easily checked by a human curator, and our approach applies such a check to the input.

### Overview of the aCM generation process

The construction of an aCM (greenish box in Figure 1) follows the ideas of constructing a classical CM with ALTERNAL. The task becomes more difficult, because we not only have to deal with several consensus structures ${SS}_{\textit {cons}}^{i}$ yielding different guide trees *g* *t*_*i*_ (indicated by the white box in Figure 1), but we also have to combine all *n* of them into one ambivalent guide forest *F*. Once *F* is found, generation of the topology defining grammar is done by evaluating *F* with a corresponding generating algebra $\mathcal {A}_{\textit {aCFG}}$. In the following sections, we give explanations about processes in aCM that have to be modified compared to ALTERNAL:

1. **Compatibility check**: Check for compatibility (defined below) of the consensus structures.
2. $\boldsymbol{\mathcal {G}}_{5s}$ **‐parsing**: Parsing each consensus structure with $\mathcal {G}_{5s}$ to gain individual guide trees.
3. **Merge**: Combine all guide trees into one ambivalent guide forest.
4. **Index**: Distribute non‐terminal indices in a depth‐first manner in the ambivalent guide forest.
5. $\boldsymbol{\mathcal {A}}_{\textit {aCFG}}$ **‐evaluation**: Generate grammar rules for the aCM by parsing the indexed ambivalent guide forest in a top‐down fashion.

### Check for compatibility of consensus structures

Two requirements are imposed on the structures in the input alignment: a) base‐pair persistence and b) global nesting. Taking the two requirements together, we call this compatibility of consensus structures.

The *base‐pair persistence* property demands that if *MSA* columns *k* and *l* are paired in consensus structure *i* those two positions must either be paired with each other in all other consensus structures, or may be unpaired, or maybe deleted. But neither *k* nor *l* are allowed to form pairs with other alignment columns than each other. For example, we think that the two consensus structures $\left \langle {\begin {array}{cc}\text {\tt {{<}\!{<}{-}}}*\text {\tt {>}}*\!*\text {\tt {>}}\\\text {\tt {{<}\!{<}{-}}}*\text {\tt {{>}{-}{-}{>}}}\end {array}}\right \rangle $can explain a common evolution up to the point where two additional unpaired bases are inserted into the upper consensus. The double gapped column $\left \langle {\!\begin {array}{cc}\text {\tt {-}}\\\text {\tt {-}}\end {array}\!}\right \rangle $ isallowed to enable inclusion of rare sequences into *MSA*, which holds insertions relative to both consensus structures. But we disallow alternative base‐pair partners, like $\left \langle {\begin {array}{cc}\text {\tt {{<}\!{<}}}\!*\!*\text {\tt {{>}\!{>}}}\\\text {\tt {<}}\!*\!\text {\tt {<}}\!*\!\text {\tt {>}\!{>}}\end {array}}\right \rangle $. Base‐pair slippage in this example must be modeled via insertions.

The *global nesting* property ensures that the multiple consensus structures do not exhibit crossing base pairs. To exclude pseudoknots in a singe secondary structure, every two base‐pairs of the structure must either be adjacent to each other or be nested within each other. With several structures, it is not sufficient that each structure satisfies this criterion. We transfer this property as *global nesting* to the alignment of *n* consensus structures by demanding that the union of all base‐pairs from all *n* consensus structures must be either adjacent or nested.

**Figure 9:**
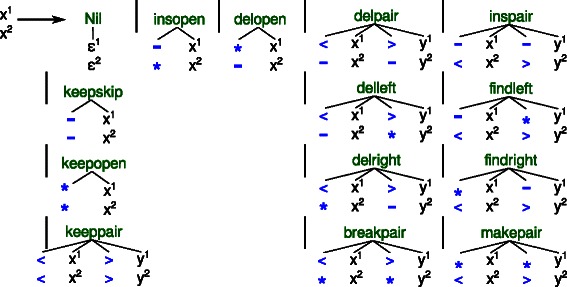
Two‐track ADP grammar $\mathcal {G}_{\textit {ali\_SS}}$ to align two consensus structures. Please note that non‐terminal *x* is composed of two tracks, *x* ^1^ and *x* ^2^.

To check if all *n* consensus structures satisfy base‐pair persistence and global nesting, we parse every $\binom {n}{2}$ pairs of two consensus structures with the two‐track ADP grammar $\mathcal {G}_{\textit {ali\_SS}}$, see Figure 9. Parsing one pair of consensus structures of length *m* with $\mathcal {G}_{\textit {ali\_SS}}$ requires a run‐time of *O*(*m* ^3^). Thus, checking all $\binom {n}{2}$ pairs needs *O*(*m* ^3^·*n* ^2^) time. Should one consensus structure pair be un‐parsable by $\mathcal {G}_{\textit {ali\_SS}}$, an error message is issued and the construction task is aborted.

### Parsing individual guide trees

**Figure 10:**
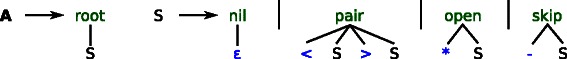
Grammar $\mathcal {G}_{5s}$ to parse a gapped consensus structure. It is identical to $\mathcal {G}_{5}$ (Figure 3), but has a forth alternative production rule to parse gap characters _.

As in classical CMs, a guide tree rules the topology of the aCM to be built. For aCMs, we individually infer one guide tree for each consensus structures. First, columns containing a majority of gaps are removed. This insertion masking process operates individually on each subfamily, the resulting ${SS}_{\textit {match}}^{i}$ can have different lengths. For example, ${SS}_{\textit {match}}^{purple}$ is one position shorter than ${SS}_{\textit {match}}^{red}$, due to a majority of gaps in column 7 for the three purple sequences. The remedy to remain the same alignment coordinates in the different consensus structures is the introduction of gaps in ${SS}_{\textit {match}}^{i}$. This is accomplished by parsing ${SS}_{\textit {match}}^{i}$ with a gapped version of $\mathcal {G}_{5s}$, see Figure 10. The resulting guide trees for both consensus structures of the example are depicted in Panel B) of Figure 8.

### Ambivalent guide forest

The *ambivalent guide forest* is a tree that encodes several guide trees. Common parts of the guide trees are shared. Where the guide trees diverge, the ambivalent guide forest contains an extra fork node, which has the diverging substructures from different guide trees as its subtrees. We need to merge all individual guide trees *g* *t*_*i*_ from the $\mathcal {G}_{5s}$‐*parsing* process in a progressive fashion into one ambivalent guide forest *F*. The progressive merge process operates top‐town.

Two guide trees for compatible consensus structures like $\left \langle {\begin {array}{cc}\text {\tt {{<}{>}}}\\ **\end {array}}\right \rangle $can differ directly at their root nodes, immediately requiring a fork node. In general, the ambivalent guide forest places the fork nodes as low as possible, to keep the aCM – and thus run‐time of the aCM – as small as possible. During construction, we also have to make sure that no unseen combinations become possible, e. g. $\left \langle {\begin {array}{cc}\text {\tt {{<}\!{<}}}\!*\!\text {\tt {>}}\!*\!\text {\tt {{-}{-}{>}}}\\ \text {\tt {{<}{-}{-}{-}}}\!*\!\text {\tt {{<}{>}\!{>}}}\end {array}}\right \rangle $shall not not allow for model structure <--- ∗ -->.

The overall idea when merging two guide trees is to overlay them and identify identical parts. When both guide trees start to diverge, a fork for both alternatives is be introduced in *F*. Identical parts are identified by simultaneously traversing both guide trees top‐down. The coupled traversal starts at both root nodes, e. g. at the red *p* *a* *i* *r*_1_ and the purple *p* *a* *i* *r*_1_ of Panel B in Figure 8. If the current nodes of both guide trees are of the same type (*nil*, *pair*, *open* or *skip*) and refer to the same alignment positions, a new node of this type is introduced in *F* (right hand side *p* *a* *i* *r*_1_ in Figure 8) and the coupled traversal is simultaneously applied to all children. If either the types or the alignment positions of the nodes under consideration differ (e. g. red *p* *a* *i* *r*_6_ and purple *o* *p* *e* *n*_6_), the complete sub‐trees, which are rooted by these nodes, are added to *F* under a fork node.

An aCM should be able to hold more than two consensus structures. Thus, it is necessary to progressively add further guide trees to *F*. The coupled traversal must be modified in the sense that a node of the guide tree is compared not to a single node in *F*, but to all guide trees already encoded in *F*, only inducing a fork if it brings in a new subtree.

The ambivalent guide forest for the red and purple guide trees of the example is depicted on the right hand side of Panel B) in Figure 8. Nodes shared by both guide trees are colored in black. The right child of *p* *a* *i* *r*_2_ is not a single tree, but a forest for the divergent red and purple sub‐trees.

The more similar a set of guide trees are, the larger are those parts of the trees that can be jointly represented in the *F*. In the worst case, there are no common parts in all *gt* at all. This means that *n* individual guide trees, each with a number of leaves that is linearly proportional to *m*, will cause a *F* with *n*·*m* nodes. Depth‐first traversal of such *F* requires *O*(*n*·*m*) time and has to be repeated during the progressive construction of *F* itself *n*‐times. Thus, the worst‐case overall run‐time for merging *n* guide trees into one ambivalent guide forest is *O*(*n* ^2^·*m*).

The construction of the ambivalent guide forest leaves room for improvement by recognizing shared substructures. The multi‐structure parsing of Saffarian *et al.* [5] makes use of shared subgrammars for shared substructures. This keeps the grammar smaller and avoids extra tables in the CYK‐type parser. Such sharing works for combinatorial matching as well as for energy minimization, as all instances of a shared subgrammar undergo the same scoring. At first glance, it appears not to be applicable with stochastic models, because shared subgrammars have their parameters trained from different sub‐families. Scoring shared substructures in different ways, of course, requires separate dynamic programming tables. However, in practice parameters may be tied together in the training phase anyway, or may come out of the training quite similar. In this situation, subgrammar sharing may be a good pragmatic decision. We leave this topic for future research.

### Provide indices in the ambivalent guide forest

We have two objectives for the design of aCM topologies: On the one hand, we want to enable alternative parses through the aCM, reflecting the ambivalent consensus structures. We have to take care that transitions are forbidden that are not indicated by the set of consensus structures: $\left \langle {\begin {array}{cc}\text {\tt {{<}\!{<}}}\!*\!\text {\tt {>}}\!*\!\text {\tt {{-}{-}{>}}}\\ \text {\tt {{<}{-}{-}{-}}}\!*\!\text {\tt {{<}{>}\!{>}}}\end {array}}\right \rangle $shall not impose model structure <--- ∗ -->. We do so by using ambivalent guide forests, which introduce branches as soon as two sub‐trees differ. This effects the allowed transitions within the model, i. e. the grammar. On the other hand, an aCM should capture as much shared alignment parts as possible: The ambivalent guide forest for $\left \langle {\begin {array}{cc}*\!*\!\text {\tt {{<}{-}{>}}}*\\ *\text {\tt {{<}\!{<}}}\!*\!\text {\tt {{>}\!{>}}}\end {array}}\right \rangle $branches into two independent sub‐trees for ∗<->∗ and << ∗ >>. However, the base‐pair from position 3 to 5 is shared between both consensus structures. Counting the emitted nucleotides should be in a sub‐family combined fashion for those positions, which are torn apart by the ambivalent guide forest. To combine both objectives, we use two types of indices: One for the non‐terminals of the grammar, affirming correct transitions and another one for the CYK algebra to share common emission probabilities. The grammar indices are given as superscripts left of the nodes for the example in Panel B) for Figure 8, while algebra indices are subscripts right of the nodes. Please note that grammar indices are unique. Algebra indices can be shared, e. g. *O*_6_ and *P*_6_. By construction and using common alignment coordinates the algebra index is directly inherited from the individual guide trees. To obtain grammar indices, we need to distribute a new set of successive numbers to the ambivalent guide forest in a depth‐first like strategy. The indexing of a list of ambivalent guide forests starts with depth‐first indexing of the complete first tree and then continues with the rest of the list.

### Generate aCM grammar

**Figure 11:**
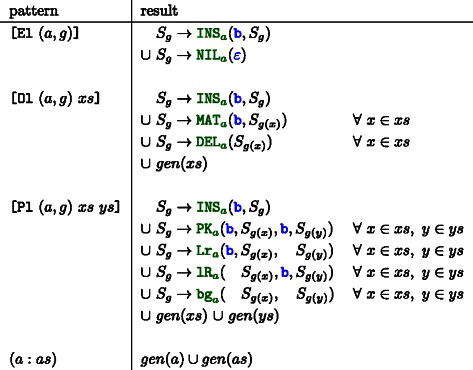
Algebra like function *gen* that evaluates a list of ambivalent guide forests into a set of grammar rules. Rules with identical indexed non‐terminals will be combined as alternative right hand sides. The function *g*(*x*) returns the grammar index of the root node of an ambivalent guide forest *x*. Index tuple (*a*,*g*) holds algebra and grammar index, respectively. Remember that *xs* and *ys* are *lists* of ambivalent guide forests.

The final step to obtain the aCM grammar is to evaluate the ambivalent guide forest with the function *gen*, given in Figure 11. A simple structural recursion on *F* performs this evaluation and returns the family model grammar. An example grammar is illustrated in Panel C) of Figure 8.

## Evaluation

We now evaluate aCMs by considering several RNA families whose structural variation can only be expressed unsatisfactorily in RFAM. We take a look at the tRNA, U5 spliceosomal RNA, RNAse‐P, and IRES families. The tRNA application is discussed in detail, where we also compare to implementing families as two separate models with classical CMs. For the others, we mainly report the results.

### The tRNA family as an aCM

Let us come back to the initial example of the tRNA family from RFAM (ID: RF00005, release 10.1). We sub‐divide the 967 sequences of the seed alignment with respect to the presence or absence of the variable fifth helix. Thus, we obtain the “cloverleaf” sub‐family with 820 members and the famous cloverleaf as its consensus structure *S* *S*_*cloverleaf*_, as stored in RFAM, as well as the sub‐family “varloop”, which holds the remaining 147 sequences. The consensus structure of “varloop” is better described by *S* *S*_*varloop*_, which contains the additional helix. The consensus *S* *S*_*varloop*_ is constructed by aligning individual structure predictions from TRNASCAN-SE [22] with RNAFORESTER [3].

We create four different classical CMs with ALTERNAL:

1. **all cloverleaf**: *MSA* contains all 967 sequences; consensus structure is *S* *S*_*cloverleaf*_. This is the original model.
2. **all varloop**: *MSA* contains all 967 sequences; consensus structure is *S* *S*_*varloop*_.
3. **only cloverleaf**: *MSA* contains 820 “cloverleaf” sequences; consensus structure is *S* *S*_*cloverleaf*_.
4. **only varloop**: *MSA* contains 147 “varloop” sequences; consensus structure is *S* *S*_*varloop*_.

**Figure 12:**
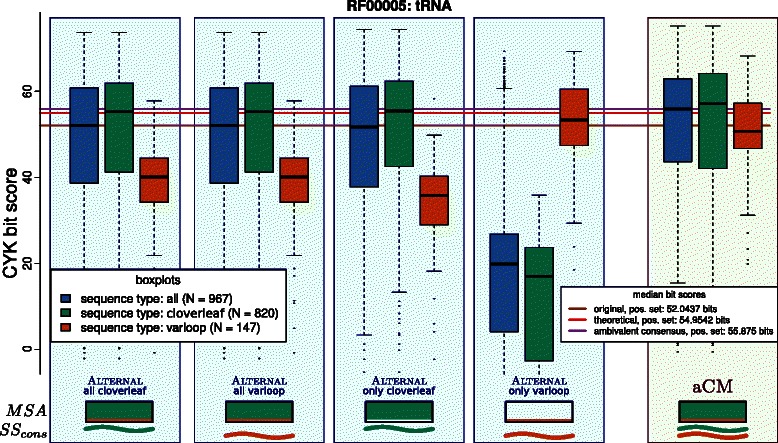
Evaluation of different ways to construct individual CMs for sub‐families for the tRNA family, compared with one aCM for both sub‐families. Detailed explanations are in the main text.

The performance of these models is shown in Figure 12. The four blue shaded columns present the results when matching all 967 members of the family to the four different CMs via the CYK algorithm. The lower part of the figure informs about the input for the model creation. The box depicts the input alignment, the curve represents the consensus structure. “cloverleaf” sequences or consensus structures are addressed in green. “varloop” components are red. There are three box‐plots in each column: The green box‐plot corresponds to those bit‐scores that stem from sequences of the sub‐family “cloverleaf” and the red box‐plot is exclusively for the “varloop” sequences. The blue box‐plot is the union of the two sub‐families and represents all bit‐scores.

The median bit‐score (brown horizontal line) for all tRNAs with the original model (leftmost blue shaded column: “all cloverleaf”) is ≈52.0. When we differentiate between both sub‐families, we see that in fact the “varloop” sequences (red box‐plot) get penalized for the additional bases of the variable loop. Their median (≈40.1) is significantly lower than the median of the green “cloverleaf” group (≈55.2). Our second CM (“all varloop”) was constructed from a different consensus structure input (*S* *S*_*varloop*_ instead of *S* *S*_*cloverleaf*_), but due to the *gap column masking* process it gives an identical guide‐tree when compared to the original “all cloverleaf” model. And indeed, we do not observe any changes in the bit‐scores.

Different *S* *S*_*match*_ and guide‐trees can only be achieved by also using different parts of the *MSA*, as done for the last two models “only cloverleaf” and “only varloop”. Training only with the cloverleaf sub‐family results in no significant shift of the overall bit‐scores (blue box‐plot); median is reduced by ≈0.3 bits. A closer look reveals that the “varloop” sequences, which are now left out for training, perform even worse (median decreases by ≈4.3 bits), while the “cloverleaf” sequences get a tiny bonus of ≈0.2 bits for their median. Good scores for “varloop” sequences can be obtained if we use the “only varloop” model. Their median is now at ≈53.3 bits. As to be expected, performance for the “cloverleaf” majority of the sequences nosedives (median at ≈17.0 bits).

Should we be able to a priori use the correct sub‐model for each sequence, or run both models in every case and cherry‐picking the better score, we would gain the theoretical median score of ≈55.0 bits (magenta horizontal line), which is ≈3.0 bits better than the original model. Splitting the family into two separate sub‐families seems to be worth it. This summarizes what can be achieved with tRNA by using classical CMs.

Applying our aCM approach relieves the creator to artificially decompose her data into two or more sub‐families in order to suit the underlying scoring algorithm. The relationship of all sequences can be kept in one alignment, now enriched by sub‐family annotations and additional consensus structures. With this evaluation setup, we expect a properly built aCM at best to return the same bit‐scores for the sequences of the various sub‐families as a cherry picking of the results from several sub‐family specific CMs would do.

The right green shaded column in Figure 12 shows the results for such an aCM for the tRNA example. It is built from both consensus structures (*S* *S*_*cloverleaf*_ and *S* *S*_*varloop*_) and trained with the total alignment (all 967 sequences). A rough inspection attests the targeted effect: A single model can both lift the “varloop” sequences to a niveau close to the overall median and simultaneously keep the good scores for the “cloverleaf” group.

Surprisingly, the aCM is doing even better than the theoretical cherry picking. As one can see by examination of the red and green box‐plots in the green shaded column of Figure 1, not only the scores for the “varloop” sequences are lifted to a reasonable amount, also the “cloverleaf” sequences benefit from this information enrichment. The median score of the aCM is ≈55.9 bits (purple horizontal line) and thus ≈0.9 bits better than the theoretical optimum. The explanation is that aCMs contain more information than a set of sub‐family specific CMs. Common sub‐structures stronger deviate from the background model and predetermined sub‐structures do not get penalized. The median score of negative sequences between the original CM and the aCM raises by +1.7 bits (data not shown). Thus, the discriminatory power (as the difference between median bit score of positive and negative test cases) can be increased by 2.1 bits, which means that even those true candidates can get positive scores, which fall below the threshold of an individual CM.

### U5 spliceosomal RNA as an aCM

**Figure 13:**
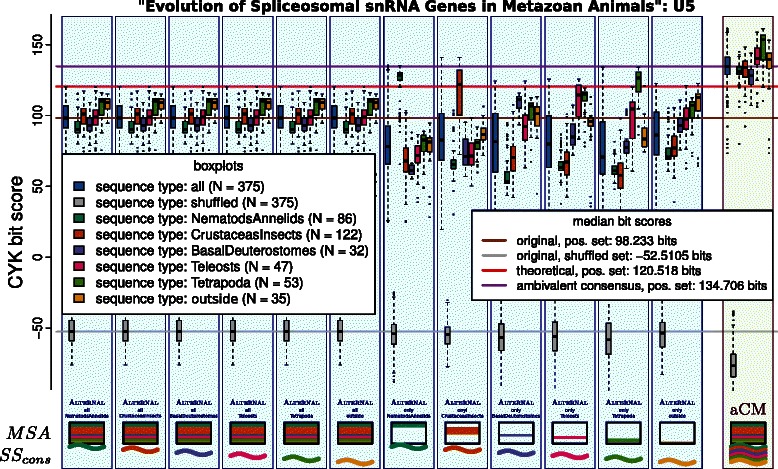
Evaluation of different ways to construct individual CMs for sub‐families for the U5 family, compared with one aCM for all six sub‐families.

An aCM can also be constructed for more than two sub‐families: The U5 spliceosomal RNA is a widespread family. Its concrete secondary structure changes over the different taxa. We used the original alignment from [23,24] identified six taxa, grouped the sequences, and defined according consensus structures. Figure 13 is the result of the same kind of analysis as shown in Figure 12; negative, i. e. di‐nucleotide shuffled sequences, are included here. In conclusion, the mean bit‐score could be increased by 27*%*, compared to the original CM, while bit‐scores for negative sequences remain low, thus improving discriminatory power.

The tRNA and U5 RNA families illustrate that the concept of aCMs can protect real families of being artificially torn apart and thus preserve existing relations. We might even render already conducted break‐ups unnecessary and merge separate families of an RFAM clan.

### Merging RNAse‐P RNA families

**Figure 14:**
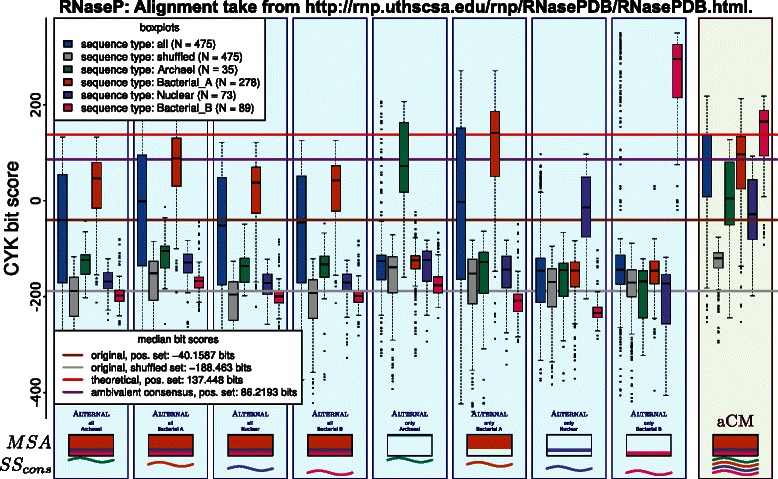
Evaluation of the effect of merging all four separate families of RNAseP.

RNAse‐P RNA is a difficult family. Despite sharing a significant structural core, some RNAs may differ locally to such a degree that their representation as one multiple sequence alignment would lead to an unmanageable “horizontal expansion”, i. e. a large proportion of gaps, as reported in [25]), and has led the curators of RFAM to construct sub‐alignments, organized in *clans*. One example is the RNAse‐P clan, which is represented in RFAM by six individual families. The success of a joint aCM for RNAse‐P heavily depends on the quality of an unifying alignment, which must capture similar regions and allowing for local structural alternatives. For a preliminary evaluation, we resort to the expertise of the authors of [26], who provide an semi‐automatically generated RNAse‐P alignment for a subset of current RFAM sequences in the clan. The sequences are organized in four sub‐groups, each with its own consensus structure. From this alignment, the aCM is generated. The results are shown in Figure 14.

While the median bit‐score of aCMs is below the theoretical optimum for cherry‐picking from individual CMs, it is much better than median bit‐scores for any combined traditional CM. The discriminatory power is improved by ≈58 bits.

There is much space for further improvement for RNAse‐P, since the used alignment has large horizontal expansions. Of the 1,669 alignment columns in total, 979 columns are masked as gap‐columns in all four sub‐groups and there are only 91 positions where all sub‐groups agree in having a match‐column.

### Merging IRES families

**Figure 15:**
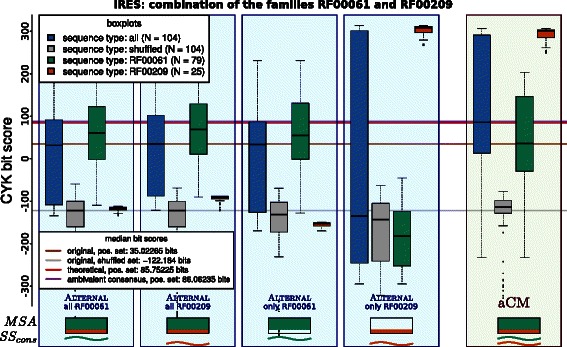
Evaluation of the effect of merging the two individual IRES families RF00061 (“Hepatitis C virus internal ribosome entry site”) and RF00209 (“Pestivirus internal ribosome entry site”) of RFAM via one aCM.

A second use‐case for the merging capabilities of aCM can be the 27 internal ribosome entry site (IRES) families of RFAM. We determined RF00061 and RF00209 as the two closest IRES families, by computing their link‐score with CMCOMPARE [27]. We then structurally aligned their consensus structure and sequence with RNAFORESTER and finally mapped the individual alignment columns to a unifying alignment with two sub‐groups. The results of our evaluation setup are given in Figure 15. Despite the poor quality of the completely automatically created alignment, the according aCM performs as well as a theoretical cherry picking of both single families.

## Conclusion

The RFAM database contains several families that cross the limitations of a single consensus structure. The new concept of an RFAM “clan” is a set of closely related families. It seems to be a workaround to cope with several consensus structures within one former family, which is now torn apart. Our evaluation shows that a segmentation of a family impedes homology analysis. Based on our implementatio and evaluation of aCMs, we suggest to extend present practice of RNA family modeling to hold multiple consensus structures to keep up with biologically motivated family definitions.

## Additional file

Additional file 1 **Supplementary material.**

## References

[1] Burge SW, Daub J, Eberhardt R, Tate J, Barquist L, Nawrocki EP, et al. Rfam 11.0: 10 years of RNA families. Nucleic Acids Res. 2012. doi:10.1093/nar/gks1005. http://nar.oxfordjournals.org/content/early/2012/11/02/nar.gks1005.full.pdf+html. 10.1093/nar/gks1005 PMC3531072 23125362

[2] Rfam Help. http://rfam.sanger.ac.uk/help, seen on March 7th, 2014.

[3] Höchsmann M, Voß B, Giegerich R (2004). Pure multiple RNA secondary structure alignments: a progressive profile approach. IEEE/ACM Trans Comput Biol Bioinform.

[4] Eddy SR, Durbin R (1994). RNA sequence analysis using covariance models. Nucleic Acids Res.

[5] Saffarian A, Giraud M, Touzet H. Proceedings of 1st Workshop on Computational Methods for Structural RNAs (CMSR’14) In: Jossinet F, Ponty Y, Waldispühl J, editors. Strasbourg, France: 2014. p. 13–24. doi:10.15455/CMSR.2014.0002. http://dx.doi.org/10.15455/CMSR.2014.0002.

[6] Reinkensmeier J, Giegerich R. Thermodynamic matchers for the construction of the cuckoo RNA family. RNA Biology 2015;12:197–207. In print http://www.tandfonline.com/doi/abs/10.1080/15476286.2015.1017206. 10.1080/15476286.2015.1017206 PMC4615179 25779873

[7] Giegerich R. Introduction to stochastic context free grammars In: Gorodkin J, Ruzzo WL, editors. RNA Sequence, structure, and function: computational and Bioinformatic methods. Methods in Molecular Biology, Humana Press: 2014. p. 85–106. doi:10.1007/978‐1‐62703‐709‐9_5. http://dx.doi.org/10.1007/978‐1‐62703‐709‐9_5. 10.1007/978-1-62703-709-9_5 24639156

[8] Durbin R, Eddy SR, Krogh A, Mitchison G. Biological sequence analysis: probabilistic models of proteins and nucleic acids: Cambridge University Press; 2002. http://www.worldcat.org/isbn/0521629713.

[9] Nawrocki EP, Eddy SR (2013). Infernal 1.1: 100‐fold faster RNA homology searches. Bioinformatics.

[10] Eddy S (2002). A memory‐efficient dynamic programming algorithm for optimal alignment of a sequence to an RNA secondary structure. BMC Bioinformatics.

[11] Weinberg Z, Ruzzo WL (2006). Sequence‐based heuristics for faster annotation of non‐coding RNA families. Bioinformatics.

[12] Nawrocki EP, Eddy SR (2007). Query‐Dependent Banding (QDB) for faster RNA similarity searches. PLoS Comput Biol.

[13] Giegerich R, Höner zu Siederdissen C (2011). Semantics and ambiguity of stochastic RNA family models. EEE/ACM Trans Comput Biol Bioinform.

[14] Dowell R, Eddy S (2004). Evaluation of several lightweight stochastic context‐free grammars for RNA secondary structure prediction. BMC Bioinformatics.

[15] Gerstein M, Sonnhammer ELL, Chothia C (1994). Volume changes in protein evolution. J Mol Biol.

[16] Krogh A, Mitchison G (1995). Maximum entropy weighting of aligned sequences of proteins or DNA,. Proc Int Conf Intell Syst Mol Biol ISMB.

[17] Johnson S. Remote protein homology detection using hidden markov models. PhD dissertation, Graduate School of Arts and Sciences of Washington University, Saint Louis, Missouri (December 2006).

[18] Giegerich R, Meyer C, Steffen P (2004). A discipline of dynamic programming over sequence data,. Sci Comput Program.

[19] Steffen P, Giegerich R (2005). Versatile and declarative dynamic programming using pair algebras. BMC Bioinformatics.

[20] Sauthoff G, Möhl M, Janssen S, Giegerich R (2013). Bellman’s GAP–a language and compiler for dynamic programming in sequence analysis. Bioinformatics.

[21] Jiang M, Anderson J, Gillespie J, Mayne M (2008). uShuffle: A useful tool for shuffling biological sequences while preserving the k‐let counts. BMC Bioinformatics.

[22] Lowe TM, Eddy SR (1997). tRNAscan‐SE: a program for improved detection of transfer RNA genes in genomic sequence. Nucleic Acids Res.

[23] Marz M, Kirsten T, Stadler PF (2008). Evolution of Spliceosomal snRNA genes in Metazoan animals. J Mol Evol.

[24] Supplemental material of Reference 23: Evolution of Spliceosomal snRNA Genes in Metazoan Animals. www.bioinf.uni‐leipzig.de/Publications/SUPPLEMENTS/08‐001/ALIGNMENTS/ALL.U5.stk. 10.1007/s00239-008-9149-6 19030770

[25] Brown JW, Birmingham A, Griffiths PE, Jossinet F, Kachouri‐Lafond R, Knight R (2009). The RNA structure alignment ontology. RNA.

[26] Zwieb C, Nakao Y, Nakashima T, Takagi H, Goda S, Andersen ES (2011). Structural modeling of RNase p RNA of the hyperthermophilic archaeon pyrococcus horikoshii {OT3}. Biochem Biophy Res Commun.

[27] Siederdissen CHz, Hofacker IL (2010). Discriminatory power of RNA family models. Bioinformatics.

