# Supplementary material for: Ambivalent covariance models
Source: BMC Bioinformatics. 2015 May 28;16:178. doi: 10.1186/s12859-015-0569-1 (PMC4504443; doi:10.1186/s12859-015-0569-1)
Supplement: Supplementary file 1 — Supplementary material. [file 12859_2015_569_MOESM1_ESM.pdf]

1 Supplement to the BMC Bioinformatics article "Ambivalent Covariance Models"

1.1 Replicating the guide-tree selection of Infernal

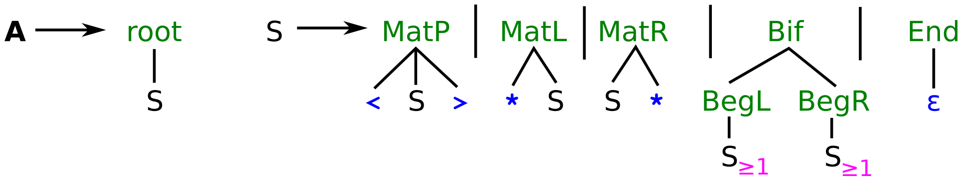

Figure 1: The syntactically ambiguous grammar $\mathcal{G}_1$ used by INFERNAL to parse SS_{match} and construct a *guide-tree*. Axiom is A.

The guide-tree (gt) determines the architecture of a CM. In INFERNAL, the syntactically ambiguous grammar $\mathcal{G}_1$ (see Figure 1) is used to parse the consensus structure SS_{match}. The result is not a single tree, but a forest $(gt_1, \ldots, gt_i, \ldots gt_n)$ from which one guide-tree (gt_i) must be chosen according to a specific set of objectives. These objectives are formulated in [1]:

> "In general there will be more than one possible guide tree for any given consensus structure. Almost all of this ambiguity is eliminated by three conventions: (1) MATL nodes are always used instead of MATR nodes where possible, for instance in hairpin loops; (2) in describing interior loops, MATL nodes are used before MATR nodes; and (3) BIF nodes are only invoked where necessary to explain branching secondary structure stems (as opposed to unnecessarily bifurcating in single stranded sequence). One source of ambiguity remains. In invoking a bifurcation to explain alignment columns $i..j$ by two sub-structures on columns $i..k$ and $k+1..j$, there will be more than one possible choice of k if $i..j$ is a multifurcation loop containing three or more stems. The choice of k impacts the performance of the divide and conquer algorithm; for optimal time performance, we will want bifurcations to split into roughly equal sized alignment problems, so I choose the k that makes $i..k$ and $k+1..j$ as close to the same length as possible."

We can precisely reformulate these objectives in ADP by a set of evaluation algebras (Table 1). A formal proof of the un-ambiguity is pending, but for each of the 2,208 RFAM (release 11.0) families just one guide-tree survived the algebra product:

$$\mathcal{P}_{select} = ((((\mathcal{A}_{noSSbif} * \mathcal{A}_{minRight}) * \mathcal{A}_{lowerBif}) * \mathcal{A}_{pushRight}) * \mathcal{A}_{balance}) * \mathcal{A}_{lighterLeft}.$$

Ordering of algebras is important. Their intentions are explained below:

- $\mathcal{A}_{noSSbif}$
  <u>Bif</u>urcations are only allowed if both children contain at least one base-pair. Thus, there cannot be <u>s</u>ingle <u>s</u>tranded regions, produced by bifurcations.

- $\mathcal{A}_{minRight}$
  Whenever an unpaired base can be modeled via a MatL or MatR, MatL is preferred. For example *** will result in three successive MatLs, not e. g. in two MatRs and one MatL. Thus, this criterion minimizes the use of right bases (MatR).

- $\mathcal{A}_{lowerBif}$
  A Bif is applied as late as possible, thus its parent is as long as possible. For example, **<><> will be MatL(*, MatL(*, Bif(BegL(MatP(<, End(), >)), BegR(MatP(<, End(), >))))) but not Bif(BegL(MatL(*, MatL(*, MatP(<, End(), >)))), BegR(MatP(<, End(), >))). Seen as a tree, Infernal lowers `Bif` as much as possible.

- $\mathcal{A}_{pushRight}$
  The use of MatR is applied as late as possible. For example, **()* will be MatL(*, MatL(*, Bif(BegL(MatP(<, End(), >)), BegR(MatR(END(), *))))) but neither MatL(*, MatR(MatL(*, MatP(<, End(), >)), *)) nor MatR(MatL(*, MatL(*, MatP(<, End(), >))), *). Seen as a tree, MatRs are pushed to the right side of the tree.

- $\mathcal{A}_{balance}$
  Whenever three or more stems have to be connected to one structure there are several ways to do so via Bif. Infernal prefers the arrangement that results in a most balanced guide-tree. The *weight* of a stem, or better a sub-tree, is the size of its yield of SS_{match}, i. e. the number of characters consumed. MatL and MatR each consume one character, while MatP consumes two; thus we can simply count those node-types in the sub-trees.

- $\mathcal{A}_{lighterLeft}$
  Should there be two balanced guide-tree candidates, the one with lighter (or equal weight) left child will be the winner.

Table 1: Set of evaluation algebras to pick the same guide-tree as INFERNAL out of the ambiguous search space, spanned by parsing SS_{match} with $\mathcal{G}_1$. min$_1$. means minimizing over the first element of a pair.

algebra function	$\mathcal{A}_{noSSbif}$	$\mathcal{A}_{minRight}$	$\mathcal{A}_{lowerBif}$	$\mathcal{A}_{pushRight}$	$\mathcal{A}_{balance}$	$\mathcal{A}_{lighterLeft}$
root(x)	x	x	$x+1$	0	x	x
MatP(a,x,b)	1	x	$x+1$	0	(x_1, x_2+2)	(x_1, x_2+2)
MatL(a,x)	x	x	$x+1$	0	(x_1, x_2+1)	(x_1, x_2+1)
MatR(a,x,b)	x	$x+1$	$x+1$	1	(x_1, x_2+1)	(x_1, x_2+1)
Bif(x,y)	$\begin{cases} -1 & \text{if } x < 1 \mid\mid y < 1 \\ 1 & \text{otherwise} \end{cases}$	$x+y$	0	0	$(\lvert x_2 - y_2 \rvert, x_2+y_2)$	$\begin{cases} (1, x_2+y_2) & \text{if } x_2 > y_2 \\ (0, x_2+y_2) & \text{otherwise} \end{cases}$
BegL(x)	x	x	x	x	x	x
BegR(x)	x	x	x	x	x	x
End(l)	0	0	1	0	$(0,0)$	$(0,0)$
$\mathcal{S}$	int	int	int	int	(int,int)	(int,int)
choice function	min.	min.	max.	min.	min$_1$.	min$_1$.

1.2 Constructing $\mathcal{G}_1$ style CMs in ADP

In the main article (Section 2.2), we described for ADP how to evaluate a guide-tree (gt) to obtain a $\mathcal{G}_5$ style context free grammar (CFG) which represents the state-to-state transitions of the CM. We can do the same for $\mathcal{G}_1$ style CMs which are used in INFERNAL.

The nomenclature of INFERNAL distinguishes between *node*s and *state*s in CMs. Each base, or base-pair of SS_{match} yields a node for the CM. The following node types are used in INFERNAL: `Root` for the start, `MatP` for a base-pair, `MatL` for an unpaired base left of some other sub-structure, `MatR` the symmetric case for a right unpaired base, `Bif` for structural bifurcations with left `BegL` and right `BegR` parts and finally a set of `End` nodes. Since there arise different situations how to align a base from the input sequence to the CM, each node consists of several states, e. g. a `MatL` nodes for an unpaired base in the model has states to match the base (`ML`), delete the base from the input (`D`) or to insert an additional base relative to the model (`IL`). Each state has a very specific set of outgoing edges to transit to the next state.

Nodes always have the following states and the associated meaning:

`Root`:
- `S`: jump to the next node.
- `IL`: insert a base left of the sub-structure.
- `IR`: insert a base right of the sub-structure.

`MatP`:
- `MP`: match both partners of the base-pair.
- `ML`: match only the left position of the base-pair, the right position is an insertion.
- `MR`: match only the right position of the base-pair, the left position is an insertion.
- `D`: both positions of the base-pair are insertions.
- `IL`: insert a base left of the sub-structure.
- `IR`: insert a base right of the sub-structure. This state is only available if the node type of the child is not `End`.

`MatL`:
- `ML`: match the unpaired position, which is left of some sub-structure.
- `D`: the unpaired position is an insertion. This state is only available if the node type of the child is not `End`.
- `IL`: insert a base left of the sub-structure.

`MatR`:
- `MR`: match the unpaired position, which is right of some sub-structure.
- `D`: the unpaired position is an insertion.
- `IR`: insert a base right to the sub-structure.

`Bif`:
- `B`: jump to children.

`BegL`:
- `S`: jump to the next node.

`BegR`:
- `S`: jump to the next node.
- `IL`: insert a base left to the sub-structure.

`End`:
- `E`: end the path through the CM.

		target node							
		Root	MatP	MatL	MatR	BegL	BegR	Bif	End
source node	Root	∅	{IL,IR,MP,ML,MR,D}	{IL,IR,ML,D}	{IL,IR,MR,D}	∅	∅	{IL,IR,B}	∅
	MatP	∅	{IL,IR,MP,ML,MR,D}	{IL,IR,ML,D}	{IL,IR,MR,D}	∅	∅	{IL,IR,B}	{IL,IR,E}
	MatL	∅	{IL, MP,ML,MR,D}	{IL, ML,D}	{IL, MR,D}	∅	∅	{IL, B}	{IL, E}
	MatR	∅	{ IR,MP,ML,MR,D}	∅	{ IR,MR,D}	∅	∅	{ IR,B}	∅
	BegL	∅	{MP, ML,MR,D}	∅	∅	∅	∅	{ B}	∅
	BegR	∅	{IL, MP,ML,MR,D}	{IL, ML,D}	∅	∅	∅	{IL, B}	∅
	Bif	Bif always transitions to both of its children BegL and BegR.							
	End	∅	∅	∅	∅	∅	∅	∅	∅

Table 2: Alternative transitions for a state x to a following state y, if x has node type "source node" and y will have node type "target node". To avoid ambiguous paths, the IL alternative is revoked if x itself is of type IR.

The scheme of state-to-state transitions within the CM automaton is reported in Table 2. The presence of a transition depends on the current source node (y-axis), its target node (x-axis) and the current state for a special case: To avoid ambiguous paths through the model, IR to IL transitions are generally forbidden.

In analogy with $\mathcal{A}_{CFG}$ of the main text, we can evaluate the given gt with the ADP algebra $\mathcal{A}_{\text{INFERNAL}}$ (see Table 3) to obtain a CFG in $\mathcal{G}_1$ style, as used by INFERNAL. The correct guide-tree can either be determined by $\mathcal{P}_{select}$ (see previous section) or decoded from the CM-file, which could be produced by CMBUILD. Sub-processes $\mathcal{G}_1$-*parsing* and *construction* from the flow-gram figure for INFERNAL can thus be replaced by the ADP instance

$$CFG = \mathcal{G}_1(\mathcal{P}_{select} * \mathcal{A}_{\text{INFERNAL}}, SS_{match}).$$

Table 3: Evaluation algebra $\mathcal{A}_{\text{INFERNAL}}$ to generate a BELLMAN'S GAP grammar for a given gt. Function *getStates* executes a look-up of the outgoing states in Table 2. Position specific index j is the position of the read symbol in the consensus structure, but for non-consuming functions Bif, BegL and BegR we need to extend the index to start and stop positions of the containing sub-word. Otherwise, uniqueness for indices cannot be guaranteed.

algebra function	$\mathcal{A}_{\text{INFERNAL}}$
root$((t,i,p))$	(root$, j, p$<br>$\cup$ start $\rightarrow$ begin(r0, S^j, r0)<br>$\cup$ $S^j \rightarrow S^j\_X^i(X^i)$<br>$\cup$ $IL^j \rightarrow IL^j\_X^i$(b, X^i)<br>$\cup$ $IR^j \rightarrow IR^j\_X^i(X^i$, b) if $t \neq$ End<br>$\setminus$ $IR^j \rightarrow IR^j\_IL^i(IL^i$, b)<br>$\forall\ X \in getStates$(root$, t$))
MatP$(a, (t, i, p), b)$	(MatP$, j, p$

Continued on next page

Continued from previous page

algebra function	$\mathcal{A}_{\text{INFERNAL}}$
	$\cup\ \mathtt{MP}^j \rightarrow \mathtt{MP}^j\_X^i(\mathtt{b}, X^i, \mathtt{b})$<br>$\cup\ \mathtt{ML}^j \rightarrow \mathtt{ML}^j\_X^i(\mathtt{b}, X^i)$<br>$\cup\ \mathtt{MR}^j \rightarrow \mathtt{MR}^j\_X^i(X^i, \mathtt{b})$<br>$\cup\ \mathtt{D}^j \rightarrow \mathtt{D}^j\_X^i(X^i)$<br>$\cup\ \mathtt{IL}^j \rightarrow \mathtt{IL}^j\_X^i(\mathtt{b}, X^i)$<br>$\cup\ \mathtt{IR}^j \rightarrow \mathtt{IR}^j\_X^i(X^i, \mathtt{b})$ if $t \neq \text{End}$<br>$\setminus\ \mathtt{IR}^j \rightarrow \mathtt{IR}^j\_\mathtt{IL}^i(\mathtt{IL}^i, \mathtt{b})$<br>$\forall\ X \in getStates(\text{MatP}, t))$
$\text{MatL}(a, (t, i, p))$	$(\text{MatL}, j, p$<br>$\cup\ \mathtt{ML}^j \rightarrow \mathtt{ML}^j\_X^i(\mathtt{b}, X^i)$<br>$\cup\ \mathtt{D}^j \rightarrow \mathtt{D}^j\_X^i(X^i)$ if $t \neq \text{End}$<br>$\cup\ \mathtt{IL}^j \rightarrow \mathtt{IL}^j\_X^i(\mathtt{b}, X^i)$<br>$\forall\ X \in getStates(\text{MatL}, t))$
$\text{MatR}(a, (t, i, p), b)$	$(\text{MatR}, j, p$<br>$\cup\ \mathtt{MR}^j \rightarrow \mathtt{MR}^j\_X^i(X^i, \mathtt{b})$<br>$\cup\ \mathtt{D}^j \rightarrow \mathtt{D}^j\_X^i(X^i)$<br>$\cup\ \mathtt{IR}^j \rightarrow \mathtt{IR}^j\_X^i(X^i, \mathtt{b})$ if $t \neq \text{End}$<br>$\forall\ X \in getStates(\text{MatR}, t))$
$\text{Bif}((t, i, p), (t', i', p'))$	$(\text{Bif}, j, p \cup p'$<br>$\cup\ \mathtt{B}^j \rightarrow \mathtt{B}^j\_\mathtt{S}^i\_\mathtt{S}^{i'}(\mathtt{S}^i, \mathtt{S}^{i'})$
$\text{BegL}((t, i, p))$	$(\text{BegL}, j, p$<br>$\cup\ \mathtt{S}^j \rightarrow \mathtt{S}^j\_X^i(X^i)$<br>$\forall\ X \in getStates(\text{BegL}, t))$
$\text{BegR}((t, i, p))$	$(\text{BegR}, j, p$<br>$\cup\ \mathtt{S}^j \rightarrow \mathtt{S}^j\_X^i(X^i)$<br>$\cup\ \mathtt{IL}^j \rightarrow \mathtt{IL}^j\_X^i(\mathtt{b}, X^i)$<br>$\forall\ X \in getStates(\text{BegR}, t))$
$\text{End}(l)$	$(\text{End}, {}_il, \mathtt{E}^{il} \rightarrow \text{Nil}())$
$\mathcal{S}$	$(string, string, string)$
choice function	id

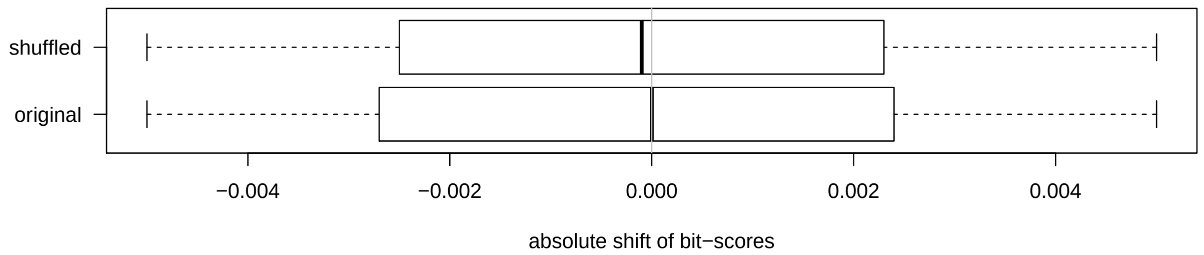

Figure 2: Bit-shift of global CYK scores between INFERNAL and upward compiled $\mathcal{G}_1$ style CMs for positive (original) and negative (shuffled) test cases in terms of absolute difference of CYK bit-scores. For example, a sequence scoring 40 bits with INFERNAL and 44 bits with ALTERNAL would have a bit-shift of +4.

1.3 CYK score deviations between Infernal and $\mathcal{G}_1$ style ADP CMs.

The main article contains a section about "Approving faithful re-implementation", where CMSEARCH results are compared to ALTERNAL results for different families f of the RFAM database. Our software ALTERNAL uses $\mathcal{G}_5$ style CMs. We here repeat the same kind of comparison, but with *upward compiled* $\mathcal{G}_1$ style CMs. The upward compiler reads the CM-file for family f, which is produced by CMBUILD, and generates $\mathcal{G}^{\mathcal{G}_1}_{f\_CM}$ and $\mathcal{A}^{\mathcal{G}_1}_{f\_CYK}$. Results are summarized in Figure 2. The mean bit-shift for the "original" sequences is 0.0000. For the "shuffled" sequences, the mean bit-shift is −0.0001. These small differences most likely stem from rounding errors, thus we conclude, that INFERNAL and our upward-compiled BELLMAN'S GAP CMs are identical.

1.4 On the inferior run-time of $\mathcal{G}_5$ vs. $\mathcal{G}_1$

The authors of [2] have shown that CMs following $\mathcal{G}_5$ are significantly smaller in terms of non-terminals and productions than $\mathcal{G}_1$. They argued that the decreased size should speed-up run-time substantially. Quite the opposite is true! The tool `multi_rt_all`, shipped with the BELLMAN'S GAP compiler, analyzes the grammar – assuming all non-terminals will be tabulated – and derives asymptotic run-times including constant factors. Table 4 exemplifies these findings with the two RFAM families covered in [2] plus – as an extreme case – family RF01960, which has the MSA with most columns in the RFAM 11.0 release. Empirical measurements (rows "real-rt") with $1,000$ random sequences with equally distributed nucleotide composition confirm the theoretical considerations of `multi_rt_all`. Note that even the asymptotic class can be reduced from $O(n^3)$ to $O(n^2)$ for $\mathcal{G}_1^{RF01380}$, if SS_{match} does not contain bifurcations. The expensive difference between both prototype grammars is that in $\mathcal{G}_5$ stretches of base-pairs, which are essentially for stable RNA structures, always bifurcate – often with empty right parts, except the potential to insert additional bases. Because of these insertions, the yield-size analysis of the BELLMAN'S GAP compiler cannot automatically correct for the expensive sub-word splits. The open question is, can we refine $\mathcal{G}_5$ to avoid unnec-

essary splits, correctly position all insertions, use fewer production rules than $\mathcal{G}_1$ and retain syntactic non-ambiguity?

Table 4: Effects of different grammar designs for identical search spaces, regarding CMs. *rules* is the number of algebra functions; *NTs* is number of non-terminals; asymptotic runtime has been determined by the tool `multi_rt_all`, shipped with the Bellman's GAP compiler. *real-rt* are real runtime measurements for *Random set 1* sequences, fitted with gnuplot via initial asymp-rt values. RF00163 and RF01380 are amongst the smallest Rfam families with and without structural bifurcations, respectively. RF01960 holds the *MSA* with most columns in Rfam 11.0. Last row is for the smaller search spaces of all trace representatives.

	family	RF00163	RF01380	RF01960
	$\lvert SS_{match} \rvert$	45	19	1,806
$\mathcal{G}_1$	rules	622	286	24,482
	NTs	143	61	5,543
	asymp-rt	$2n^3 + 1754n^2 + 18n + 19$	$790n^2 + 18n + 19$	$60n^3 + 66060n^2 + 95$
	real-rt	$-4.5^{-e8}n^3 + 2.7^{-e4}n^2 - 4.1^{-e2}n + 2.3$	$3.8^{-e5}n^2 + 9.4^{-e3}n - 1.2$	–
$\mathcal{G}_5$	rules	152	67	5,870
	NTs	47	21	1,808
	asymp-rt	$168n^3 + 315n^2 + 2$	$84n^3 + 135n^2 + 2$	$5376n^3 + 12667n^2 + 2$
	real-rt	$250^{-e8}n^3 + 7.9^{-e4}n^2 - 30.5$	$6.3^{-e7}n^3 + 76^{-e5}n^2 - 30.8$	–

2 References

[1] Sean Eddy. A memory-efficient dynamic programming algorithm for optimal alignment of a sequence to an rna secondary structure. *BMC Bioinformatics*, 3(1):18, 2002.

[2] Robert Giegerich and Christian Höner zu Siederdissen. Semantics and Ambiguity of Stochastic RNA Family Models. *IEEE/ACM Transactions on Computational Biology and Bioinformatics*, 8:499–516, 2011.
